# Polymorphic Variants of Neurotrophic Factor Genes in Affective Disorders: Pilot Study

**DOI:** 10.3390/ijms26167982

**Published:** 2025-08-19

**Authors:** Ekaterina V. Mikhalitskaya, Natalya M. Vyalova, Diana Z. Paderina, Olga V. Roschina, German G. Simutkin, Nikolay A. Bokhan, Svetlana A. Ivanova

**Affiliations:** 1 Mental Health Research Institute, Tomsk National Research Medical Center, Russian Academy of Sciences, Tomsk 634014, Russia; natarakitina@yandex.ru (N.M.V.); osmanovadiana@mail.ru (D.Z.P.); roshchinaov@yandex.ru (O.V.R.); ggsimutkin@gmail.com (G.G.S.); bna909@gmail.com (N.A.B.); ivanovaniipz@gmail.com (S.A.I.); 2 Psychiatry, Addictology and Psychotherapy Department, Siberian State Medical University, Tomsk 634050, Russia

**Keywords:** neurotrophic factors, single-nucleotide polymorphisms, SNP, genetic association, affective disorders

## Abstract

Neurotrophic factors are regulatory proteins of nervous tissue. They have a significant effect on all processes in neurons. Neurotrophic factors participate in the processes of neuronal differentiation, neurogenesis, synaptogenesis, and the regulation of neuronal plasticity. Numerous data in the literature indicate that disruption of the regulation of neurotrophic factors can play an important role in the etiology of affective disorders. We examined 235 patients with an affective disorder (F31, F32, F33, F34.1, ICD-10) and 187 healthy controls. The mental state of the patients was assessed by SIGH-SAD, HARS, and CGI scales. Genotyping of polymorphic variants of neurotrophic factor genes (*BDNF*, *NGF*, and *NRG1*) was performed using real-time PCR. Only one of the polymorphisms (rs7124442 in the *BDNF* gene) showed an association with the affective disorders. All polymorphisms (except rs11030104 in the *BDNF* gene) showed an association or associated trend with clinical characteristics of the disease, evaluated according to psychometric scales and response to therapy. Our results support the potential value of the studied neurotrophic genes as a neurobiological marker for depression pathogenesis, clinical characteristics, and response to treatment. Due to several limitations, further case–control studies with larger sample sizes and different ethnic groups are needed.

## 1. Introduction

Neurotrophic factors are considered to play a crucial role in synapse formation. The neurotrophin family of highly conserved proteins includes brain-derived neurotrophic factor (BDNF), nerve growth factor (NGF), neurotrophin 3 (NT3), and neurotrophin 4 (NT4) [1]. Neurotrophic factors regulate structural, synaptic, and morphological plasticity to modulate the intensity or number of synaptic connections and neurotransmission. The role of neurotrophic factors in the regulation of neuronal growth and differentiation has been shown, as well as their involvement in the plasticity and survival of neurons and glia [2,3,4,5,6,7].

For example, in the adult brain, BDNF shows high levels of expression and regulates both excitatory and inhibitory synaptic transmission [8], and deficiency of BDNF signaling determines its role in the pathogenesis of a number of brain diseases and mental disorders, such as Huntington’s disease, Alzheimer’s disease, ischemic stroke [9,10], stress-related disorders [11,12], substance abuse [13,14], eating disorders, mood disorders [15,16,17], and schizophrenia [18,19]. At the same time, BDNF enhances neuronal excitability, and its expression is increased in brain areas involved in epileptogenesis.

Animal models and clinical studies provide strong evidence that dysregulation of neurotrophic factors may also play an important role in the etiology of bipolar disorder (BD) and major depressive disorder (MDD) [20]. The neurotrophic theory of depression development best explains the morphological changes in the brain that occur in affective disorders (ADs) [21,22,23].

In recent years, the possibility of using serum BDNF concentrations and/or studying *BDNF* gene polymorphisms as indicators of neuroplasticity and as potential peripheral biomarkers of depression and patients’ response to antidepressant therapy has been widely explored [24,25]. A large number of studies show that depression is associated with decreased BDNF production, which may indicate the importance of this neurotrophic factor in the etiology of depressive disorders [15,16,26,27]. In contrast, the response to antidepressant treatment is accompanied by an increase in *BDNF* mRNA levels in the hippocampus and prefrontal cortex [28]; an increase in serum BDNF concentrations is observed in individuals with depression undergoing psychotherapy or pharmacotherapy [27,29], which also correlates with improved memory and learning and with a reduction in the symptoms of this disorder [30].

Genes encoding BDNF and its associated kinases are often pharmacogenetic markers in personalized psychiatry and allow for an objective assessment of the response to pharmacotherapy with antipsychotic and antidepressant drugs and the prediction of the risk of developing side effects [31,32,33,34,35].

Genetic studies link *BDNF* gene polymorphism to depression [36]. G.H. Hosang et al. (2014) note that the *BDNF* gene may contain an impressive number of polymorphisms that may also influence the development of depression under unfavorable environmental conditions [37].

It is now considered that NGF may also be involved in the pathogenesis of depression [3,38,39,40]. Significantly lower levels of NGF have been observed in patients with MDD compared to a control group [25,41,42]. In rats, NGF administration ameliorates depressive-like behavior and alters *DRD5*, *PROKR1*, *HTR3A*, *CHRNA5*, *MAOA*, *CHRNB4*, *SSTR3*, and *CCKAR* gene expression in the amygdala and hippocampus [43]. In addition, preclinical and clinical models of depression have shown that antidepressants can control depression via NGF [25].

Another member of the neurotrophin family is NRG1 (Neuregulin 1), which interacts with the receptor tyrosine kinases, playing a role in neurodevelopment, neuronal migration, cell growth, and homeostasis of brain activity [44,45]. It has been shown that NRG1 deficiency or aberrant NRG1 signal transmission in cortical projection neurons leads to increased inhibitory connections and decreased synaptic plasticity [46]. Serum NRG-1 levels are significantly lower among depressed patients compared with controls [47], and *NRG1* SNPs (single-nucleotide polymorphisms) are associated with depression and BD [44].

Thus, neurotrophic factors play an important role in physiological and pathological processes of neuroplasticity. They regulate a number of the most important cellular functions of neurons in the brain, such as growth, differentiation, and survival, which allows an individual to carry out certain behavioral reactions. Most studies in the literature are devoted to determining the frequency of the rs6265 polymorphism in the *BDNF* gene in mental disorders, while there are only a few studies regarding other polymorphisms of this gene and other neurotrophic factor genes. The potential associations of six polymorphisms were investigated, including the association of rs7124442 with suicide in a Slavic population [48]; the role of *BDNF* SNPs (rs6265, rs16917204, rs7103411, and rs11030104) in the risk of alcohol use disorder [49]; the effect of *NGF* (rs6330) in patients with major depressive disorder [40]; and the association of *NRG1* (rs3924999) with the risk of schizophrenia [50] and with response to therapy in patients with major depression who have received selective serotonin reuptake inhibitor treatment for a period of 6 weeks [51]. Since our previous studies [17,52] showed an association of the functional SNP rs6265 in the *BDNF* gene with depression and response to therapy, we decided to extend the investigation and test additional SNPs in the *BDNF* gene, *NGF*, and *NRG1*.

## 2. Results

### 2.1. Study of Polymorphic Variants of Neurotrophic Factor Genes in Patients with AD

The data on the frequency distribution of genotypes (*BDNF* (rs7124442, rs11030104, rs7103411), *NGF* (rs6330), and *NRG1* (rs3924999) genes) were tested for compliance with HWE (Hardy–Weinberg equilibrium). All polymorphic variants were consistent with equilibrium (*p* > 0.05).

A comparative analysis of the genotype distribution frequencies of the genes of the neurotrophic factors *BDNF*, *NGF*, and *NRG1* in the AD group and HC (healthy control) group was carried out. The comparison of the studied groups revealed statistically significant differences in the frequency distribution of the *BDNF* rs7124442 alleles between the group of patients with AD and the HC group (χ^2^ = 6.465, *p* = 0.039) (Table 1). The CC*rs7124442 genotype was significantly less common in the AD group than in the HC group (2.6% and 8%, respectively, OR [95% CI] = 0.32 [0.12–0.86]). After correction for multiple comparisons (corrected statistical significance: *p* = 0.001), statistical significance disappeared. Post hoc power analysis revealed 98% power (α = 0.01, df = 2) for detecting the observed effect size (Cohen’s *w* = 0.25) for the associated SNPs, given the sample size of 412. No statistically significant differences were found for the other polymorphic variants in the studied genes (*p* > 0.05).

### 2.2. Association of Polymorphic Variants of Neurotrophic Factor Genes with Clinical Characteristics of AD

The analysis of the clinical characteristics of AD depending on the patient carrying the rs7124442 polymorphic variant of the *BDNF* gene showed a number of associations (Table 2). On day 14 of therapy, patients homozygous for the minor allele rs7124442*CC of *BDNF* had higher scores for the severity of anxiety symptoms (14.5 (9.25:19.5)), assessed by the HARS, and typical depressive symptoms (13 (10.25:15.75)), assessed by the SIGH-SAD scale, compared to carriers of the CT (7 (4.5:12.5) and 9 (5.25:12), respectively) and TT (10 (7:15) and 11 (8:14), respectively) genotypes (*p* = 0.008 and *p* = 0.019). Also, carriers of rs7124442*CC showed a tendency to have higher scores on the Clinical Global Impression scale (CGI-S) on day 28 of therapy (2.5 (2:3)), compared with carriers of the CT (2 (2:2)), and TT (2 (2:3)) genotypes (*p* = 0.072). Thus, despite the fact that the CC genotype is less common in the group of patients with AD, carrying this genotype is a risk factor for worse therapy dynamics.

When analyzing the clinical characteristics of AD (Table 3), it was shown that carriers of the rs7103411*CC *BDNF* gene had a tendency to score more highly on the Clinical Global Impression scale (CGI-S) on day 28 of therapy (2 (2:3.5)) compared to carriers of the CT (2 (2:3)), and TT (2 (2:2)) genotypes (*p* = 0.057).

When studying the associations of the clinical characteristics of AD with carrying the polymorphic variant rs11030104 of the *BDNF* gene, no statistically significant differences were found (Table 4).

An association was shown between the polymorphic variant rs3924999*AA of the *NRG1* gene and a lower score for typical depressive symptoms assessed on the SIGH-SAD scale before pharmacotherapy (Table 4) than in carriers of the AG and GG genotypes (16 [14; 21.5], 21.5 [18; 26], and 21 [17; 26], respectively, χ^2^ = 7.969, *p* = 0.019), and an association between the rs3924999*AG variant and a significantly lower score on the CGI-I scale on the 28th day of therapy was shown compared to carriers of the AA and GG genotypes (2 [1; 2], 2 [2; 2], and 2 [2; 2], respectively, χ^2^ = 9.680, *p* = 0.008). Also, carriers of the rs3924999*AA variant showed a tendency towards having a lower total score on the SIGH-SAD scale before pharmacotherapy (22 (20:32)) compared with carriers of the AG (29 (23:35.75)) and GG (29.5 (23:34)) genotypes (*p* = 0.072).

Carriers of the rs6330*AA variant of *NGF* showed a tendency towards having a higher SIGH-SAD score on the 14th day of therapy (15 (12.25:18)) compared to carriers of the AG (14 (10.5:18.75)) and GG (13 (9:15)) genotypes (*p* = 0.076) (Table 5).

## 3. Discussion

Polymorphisms of neurotrophic factor genes are very important factors to consider as they can alter the neuroplasticity function of the brain, causing multiple human phenotypes [53], and they may play a key role as a predisposing factor to mental diseases, including affective disorders [54]. In this study, we examined the possible contribution of five genetic polymorphisms in three genes encoding neurotrophic factors, namely BDNF, NGF, and NRG1, to a predisposition to the number of depressive symptoms in patients with AD in a Russian population. The novelty of our research is the study of the associations of polymorphic variants of neurotrophic factor genes with affective disorders from the point of view of the clinical manifestations of the disease, assessed by a number of psychometric scales used to determine the dynamics of therapy. Most studies in the literature focus on the determination of the frequencies of the rs6265 polymorphism in the *BDNF* gene. Our study is the first to compare the frequencies of other polymorphisms in this gene and in genes of other neurotrophic factors between patients with depression and a control group and to identify possible associations with clinical characteristics and response to antidepressant therapy.

In studies on different populations, data on the association of the polymorphic variant rs7124442 in the *BDNF* gene are contradictory. In our study, we showed that the polymorphism rs7124442 is associated with affective disorders. The *BDNF* rs7124442 polymorphism could alter the binding of miR-922 to positively regulate *BDNF* expression, thus providing a post-transcriptional mechanism for gene regulation [55]. At the same time, different independent groups of scientists have failed to show an association between AD and rs7124442 in the *BDNF* gene in German and Korean populations [56,57,58]. A study of 72 German women with fibromyalgia syndrome showed an association between rs7124442 and anxiety symptoms, but this association was not observed in the depression subgroup [59]. This heterogeneity of data may be due to the ethnic characteristics of the subjects or the small size of groups in the study. Furthermore, the frequency of the minor allele of rs7124442 in the HapMap database is 0.33, but our study showed lower frequencies both in the HC and AD groups. The other polymorphic variants in our study did not show an association with AD, which is supported by the results of a number of studies [57,60].

Our previous studies [17,52] showed an association of one polymorphic variant in the *BDNF* gene (rs6265) with treatment response. In the present study, all polymorphisms (except rs11030104 in the *BDNF* gene) showed an association or trend of association with the clinical characteristics of the disease evaluated according to psychometric scales, such as SIGH-SAD, CGI, and HARS. Calabrò M. et al., 2018, show that the polymorphic variant rs11030104 in the *BDNF* gene is not significantly associated with response, remission, or resistance to antidepressant treatment in MDD, and it was also not significantly associated with response to remission with mood stabilizer treatment in BD patients [58]. This data is consistent with the results of our study.

In addition, we have shown the association of the rs7124442*CC variant of the *BDNF* gene with worse treatment outcomes after 14 and 28 days of therapy, while a study by Domschke et al., 2010, involving a German sample showed the opposite result after 6 weeks of treatment in patients [56]. This may be due to differences between the examined group and the duration of patient observation.

**Limitations of the study:** Our research has several limitations. It is a pilot study, which is a small-scale preliminary study conducted to check the feasibility of or improve the research design and attract the attention of other researchers to a topic. The main limitation is the small size of the subgroups, which can reduce statistical power and increase the risk of Type I and II errors. Due to the small sample size, the statistical significance between the patient and control groups after Bonferroni correction disappeared. Our findings are preliminary and therefore require replication of the obtained data for other cohorts. The second limitation is the sex distribution bias: the patient group consisted mainly of women, but the HC group was only half female. The third limitation is the use of different antidepressants in therapy, which could have influenced the effectiveness of therapy and differences in responses. To overcome this limitation, longitudinal studies of patients receiving antidepressant monotherapy would be needed.

The fact that only Caucasians were included in this study limits its generalizability, although we attempted to achieve ethnic homogeneity in the sample.

**Conclusion:** Our results support the potential value of the studied neurotrophic genes as a neurobiological marker for depression pathogenesis, clinical characteristics, and response to treatment. Further case–control studies with larger sample sizes and different ethnic groups are needed.

## 4. Materials and Methods

### 4.1. Design

The study compared the distribution of genotype frequencies in two groups: patients with AD according to the ICD-10 criteria and the healthy control group (HC). In the AD group, the association of the polymorphic variants of the studied genes with the clinical characteristics of the disease was studied. Patients were examined through a range of psychometric scales upon admission, before the start of active psychopharmacotherapy, and for a month afterwards at two-week intervals. The study was conducted in accordance with the Code of Ethics of the World Medical Association and approved by the Bioethical Committee of the Mental Health Research Institute of the Tomsk National Research Medical Center of the Russian Academy of Sciences (Protocol 164, approval on 16 June 2023). All participants provided written informed consent.

### 4.2. Examined Groups

The study subjects were patients at the psychiatric departments of the Mental Health Research Institute, Tomsk National Research Medical Center, of Slavic ethnicity with a current affective episode diagnosed in accordance with the ICD-10 criteria within the framework of mood disorders: F31, F32, F33, or F34.1. The study sample consisted of 235 patients, the majority of whom were women (79.1%), and 187 healthy controls, 50.3% of whom were women.

The median age in the group of patients was 45 [34:54] years, and in the HC group, it was 39 [29.5:51] years. During the treatment process, patients received personalized therapy according to their diagnosis, clinical picture, and leading symptoms; the most frequent drugs of choice were antidepressants from the selective serotonin reuptake inhibitor group (SSRI)—52.6%; antidepressants with a polymodal mechanism of action—15.4%; or normotimics—21.5%. The median age at which symptoms of an affective disorder appeared was 30.5 (18.75:46.5) years, and the median duration of the current affective episode was 5 (3:12) months.

#### 4.2.1. Inclusion and Exclusion Criteria

Screening for relevant pathology for the inclusion/exclusion of subjects was performed through a clinical assessment on the first day of admission to the affective disorder department of the Mental Health Research Institute of the Tomsk National Research Medical Center.

Inclusion criteria: 1. Patient consent for the study; 2. established diagnosis of affective disorder in accordance with the ICD-10 criteria; and 3. age of 18–60 years.

Exclusion criteria: 1. Patient refusal to participate at any stage of the study; 2. dementia; 3. intellectual disability; 4. other severe organic diseases of the brain with pronounced cognitive impairment (encephalitis, meningitis, consequences of TBI, etc.); 5. alcohol psychosis; 6. chronic decompensated somatic diseases; 7. acute somatic diseases; 8. non-Caucasian physical appearance (e.g., Asian, Buryats or Khakassians).

#### 4.2.2. Clinical, Dynamic, and Psychopathological Examination

Clinical–dynamic and psychopathological assessment of the patients’ conditions was carried out using psychometric instruments—the Structured Interview Guide for the Hamilton Depression Rating Scale, Seasonal Affective Disorder Version (SIGH-SAD) [61], to assess the severity of depressive symptoms, taking into account atypical depressive symptoms; the Hamilton Anxiety Rating Scale (HARS) [62]; and the Clinical Global Impression scale (CGI)—upon admission and after 2 and 4 weeks of therapy [63].

The diagnosis of affective disorders in the group was represented by the following conditions: bipolar disorder (BD), F31—33.2% (n = 78); a single depressive episode, F32—30.6% (n = 72); recurrent depressive disorder, F33—25.5% (n = 60); and dysthymia, F34.1—10.6% (n = 25).

Upon admission to the clinic, the severity of depressive symptoms according to the SIGH-SAD scale was 29 (22:35) points (21 (16:26)—typical depressive symptoms; 6 (4:9)—atypical depressive symptoms), and after 4 weeks, it was 6 (3:10), 4 (2:7), and 2 (0:3) points, respectively. During therapy, a significant decrease in the anxiety level was noted according to the HARS: 18 (12:26) points upon admission and 4 (2:7) after 4 weeks of therapy. The patients’ conditions on the CGI-S scale were assessed as “moderately ill”, equivalent to 4 (4:5), upon admission and “borderline mentally ill”, equivalent to 2 (2:3), after 4 weeks of therapy. The dynamics of the conditions according to the CGI-I scale during treatment were characterized as “much improved” after 4 weeks of therapy.

### 4.3. Genetic Analysis

This study was conducted on the basis of the “Medical genomics” Core Facility.

DNA was isolated according to the standard phenol–chloroform method. Polymorphic variants of the neurotrophic factor genes *BDNF* (rs7124442, rs11030104, rs7103411), *NGF* (rs6330), and *NRG1* (rs3924999) were selected for genotyping. The following criteria were used as a strategy to choose SNPs:

1. A minor allele frequency (MAF) of at least 5%;
2. Availability of information from previous studies on a given SNP;
3. Marker localization.

Genotyping of the polymorphic variants of the neurotrophic factor genes *BDNF* (rs7124442, rs 11030104, rs7103411), *NGF* (rs6330), and *NRG1* (rs3924999) was performed by real-time PCR on a QuantStudio™ 5 Real-Time PCR System (Applied Biosystems, Waltham, MA, USA) using primers and TaqMan1 Validated SNP Genotyping Assay kits (Applied Biosystems, Waltham, MA, USA). Each assay consisted of unlabeled forward and reverse primers and FAM- and VIC-dye-labeled MGB probes. The reaction was carried out in duplicate samples using a negative control and passive reference ROX. Samples with call rates less than 90% were excluded from further analysis.

### 4.4. Statistical Analysis

Statistical processing of the obtained results was carried out in the R 3.6.2 software environment, using the R-Studio IDE. Standard functions and additional packages were used, such as “dplyr”, “tidyverse”, “psych” and “SNPassoc”.

The obtained data were preliminarily checked for compliance with Hardy–Weinberg equilibrium (HWE). Association analysis was conducted using the chi-square criterion and Bonferroni correction. Quantitative data were checked for a normal distribution, and the results showed that their distribution differed from normal. This led to the use of nonparametric statistical criteria: Mann–Whitney and Kruskal–Wallis. A significance level of less than 0.05 was used. Full data are presented in Tables S1–S6 of the Supplementary Materials.

## Figures and Tables

**Table 1:** Comparison of genotype and allele frequencies of polymorphic variants of the studied genes between the HC, and AD groups.

SNP	Genotypes/Alleles	HC Group; n, (%)	AD Group; n, (%)	OR [95% CI]	χ^2^	*p*
rs7124442 *BDNF*	TT	102 (54.5%)	127 (55.7%)	1.05 [0.71–1.55]	6.465	0.039 *
	CT	70 (37.4%)	95 (41.7%)	1.09 [0.73–1.63]		
	CC	15 (8%)	6 (2.6%)	0.32 [0.12–0.86]		
	T	0.733	0.765	1.19 [0.87–1.63]	1.176	0.278
	C	0.267	0.235	0.84 [0.61–1.15]		
rs11030104 *BDNF*	AA	128 (68.8%)	147 (66.2%)	0.89 [0.59–1.35]	0.737	0.692
	AG	55 (29.6%)	69 (31.1%)	1.09 [0.71–1.67]		
	GG	3 (1.6%)	6 (2.7%)	1.74 [0.43–7.1]		
	A	0.836	0.818	0.88 [0.61–1.27]	0.480	0.489
	G	0.164	0.182	1.14 [0.79–1.64]		
rs7103411 *BDNF*	TT	128 (69.9%)	148 (66.7%)	0.86 [0.56–1.31]	1.231	0.540
	CT	52 (28.4%)	67 (30.2%)	1.11 [0.72–1.72]		
	CC	3 (1.6%)	7 (3.2%)	2.02 [0.51–7.97]		
	T	0.842	0.818	0.84 [0.58–1.22]	0.810	0.368
	C	0.158	0.182	1.18 [0.82–1.72]		
rs6330 *NGF*	GG	64 (34%)	65 (28.6%)	0.78 [0.51–1.18]	1.606	0.448
	AG	94 (50%)	119 (52.4%)	1.25 [0.8–1.93]		
	AA	30 (16%)	43 (18.9%)	1.41 [0.79–2.52]		
	G	0.590	0.548	0.84 [0.64–1.11]	1.476	0.224
	A	0.410	0.452	1.19 [0.9–1.56]		
rs3924999 *NRG1*	GG	75 (42.9%)	82 (35.8%)	0.74 [0.5–1.11]	3.587	0.166
	AG	88 (50.3%)	121 (52.8%)	1.26 [0.83–1.91]		
	AA	12 (6.9%)	26 (11.4%)	1.98 [0.93–4.2]		
	G	0.680	0.622	0.78 [0.58–1.04]	2.896	0.089
	A	0.320	0.378	1.29 [0.96–1.73]		

The rs7124442 SNP of *BDNF* was significantly associated with AD diagnosis. AD—patients with affective disorder; HC—healthy control; OR [95% CI]—odds ratio and 95% confidence interval; SNP—single-nucleotide polymorphism; χ^2^—chi-square criterion; *—statistical significance at *p* < 0.05.

**Table 2:** Association of the polymorphic variant rs7124442 of the *BDNF* gene with the clinical characteristics of affective disorders.

Psychometric Scales	Day of Therapy	CC	CT	TT	χ^2^	*p*
HARS	upon admission	15.5 (11.25:26.5)	17 (11:26)	19 (14:26)	3.610	0.164
	on day 14 of therapy	14.5 (9.25:19.5)	7 (4.5:12.5)	10 (7:15)	9.710	0.008 *
	on day 28 of therapy	5.5 (2.75:6)	3 (2:7)	4 (2:7)	1.110	0.574
SIGH-SAD	upon admission	21 (13.25:25)	20 (16:25)	21 (17:27)	2.578	0.275
	on day 14 of therapy	13 (10.25:15.75)	9 (5.25:12)	11 (8:14)	7.893	0.019 *
	on day 28 of therapy	6 (4.25:7.75)	4 (2:7)	4 (2:7)	2.286	0.319
CGI-S	upon admission	4.5 (4:5.75)	4 (4:4)	4 (4:4.75)	3.923	0.141
	on day 14 of therapy	3 (3:5)	3 (3:3)	3 (3:4)	3.832	0.147
	on day 28 of therapy	2.5 (2:3)	2 (2:2)	2 (2:3)	5.254	0.072

The polymorphic variant rs7124442 of the *BDNF* gene was significantly associated with the severity of anxiety symptoms and typical depressive symptoms. χ^2^—chi-square criterion; *—statistical significance at *p* < 0.05.

**Table 3:** Association of the polymorphic variant rs7103411 in the *BDNF* with the clinical characteristics of affective disorders.

Psychometric Scales	CC	CT	TT	χ^2^	*p*
CGI-S upon admission	4 (4:4.5)	4 (4:5)	4 (4:4)	0.168	0.919
CGI-S on day 14 of therapy	3 (3:3)	3 (3:3)	3 (3:4)	0.369	0.832
CGI-I on day 14 of therapy	3 (3:3)	3 (2:3)	3 (2:3)	1.335	0.513
CGI-S on day 28 of therapy	2 (2:3.5)	2 (2:3)	2 (2:2)	5.723	0.057
CGI-I on day 28 of therapy	2 (1.5:2.5)	2 (1:2)	2 (1:2)	0.832	0.660

No statistically significant associations were found for the rs7103411 polymorphic variant with the clinical characteristics of AD. χ^2^—chi-square criterion.

**Table 4:** Association of the polymorphic variant rs3924999 of *NRG1* with the clinical characteristics of affective disorders.

Psychometric Scales	Day of Therapy	AA	AG	GG	χ^2^	*p*
SIGH-SAD for typical depressive symptoms	upon admission	16 (14:21.5)	21.5 (18:26)	21 (17:26)	7.969	0.019 *
	on day 14 of therapy	8 (6:10)	11 (7:13.25)	11 (8:13)	4.537	0.103
	on day 28 of therapy	4 (3:6)	4 (2:7)	4 (2:7)	0.010	0.995
CGI-I	on day 14 of therapy	3 (2.25:3)	3 (2:3)	3 (2:3)	2.107	0.349
	on day 28 of therapy	2 (2:2)	2 (1:2)	2 (2:2)	9.680	0.008 *

The polymorphic variant rs3924999 of the *NRG1* gene was significantly associated with typical depressive symptoms and the severity of AD. χ^2^—chi-square criterion; *—statistical significance at *p* < 0.05.

**Table 5:** Association of the polymorphic variant rs6330 of *NGF* with the clinical characteristics of affective disorders.

Psychometric Scales	Day of Therapy	AA	AG	GG	χ^2^	*p*
SIGH-SAD total	upon admission	29 (24.5:33.5)	30 (22:36)	28 (22:33.5)	2.123	0.346
	on day 14 of therapy	15 (12.25:18)	14 (10.5:18.75)	13 (9:15)	5.167	0.076
	on day 28 of therapy	7 (3:11)	6 (3:11)	6 (3:9)	1.391	0.499

No statistically significant associations were found for the rs6330 polymorphic variant with the clinical characteristics of AD. χ^2^—chi-square criterion.

## Data Availability

The original contributions presented in this study is included in the article Supplementary Material. Further requests concerning additional information or minimal dataset can be directed to the corresponding author.

## Supplementary Materials

The following supporting information can be downloaded at: https://www.mdpi.com/article/10.3390/ijms26167982/s1.

## Abbreviations

The following abbreviations are used in this manuscript:

AD	Affective Disorder
BD	Bipolar Disorder
BDNF	Brain-Derived Neurotrophic Factor
HC	Healthy Control
MDD	Major Depressive Disorder
NGF	Nerve Growth Factor
NRG1	Neuregulin 1
SNP	Single-Nucleotide Polymorphism
