# Supplementary material for: Polymorphic Variants of Neurotrophic Factor Genes in Affective Disorders: Pilot Study
Source: Int J Mol Sci. 2025 Aug 19;26(16):7982. doi: 10.3390/ijms26167982 (PMC12386679; doi:10.3390/ijms26167982)
Supplement: Supplementary file 1 [file ijms-26-07982-s001.zip › ijms-3764149-supplementary.pdf]

Table S1. Comparison of genotype and allele frequencies of polymorphic variants of the studied genes between the HC and AD group.

SNP	Genotypes/ alleles	HC group; n, (%)	AD group; n, (%)	OR [95% CI]	χ2	p
rs7124442 *BDNF*	TT	102 (54.5%)	127 (55.7%)	1.05 [0.71 - 1.55]	6.465	0.039*
	CT	70 (37.4%)	95 (41.7%)	1.09 [0.73 - 1.63]		
	CC	15 (8%)	6 (2.6%)	0.32 [0.12 - 0.86]		
	T	0.733	0.765	1.19 [0.87 - 1.63]	1.176	0.278
	C	0.267	0.235	0.84 [0.61 - 1.15]		
rs11030104 *BDNF*	AA	128 (68.8%)	147 (66.2%)	0.89 [0.59 - 1.35]	0.737	0.692
	AG	55 (29.6%)	69 (31.1%)	1.09 [0.71 - 1.67]		
	GG	3 (1.6%)	6 (2.7%)	1.74 [0.43 - 7.1]		
	A	0.836	0.818	0.88 [0.61 - 1.27]	0.480	0.489
	G	0.164	0.182	1.14 [0.79 - 1.64]		
rs7103411 *BDNF*	TT	128 (69.9%)	148 (66.7%)	0.86 [0.56 - 1.31]	1.231	0.540
	CT	52 (28.4%)	67 (30.2%)	1.11 [0.72 - 1.72]		
	CC	3 (1.6%)	7 (3.2%)	2.02 [0.51 - 7.97]		
	T	0.842	0.818	0.84 [0.58 - 1.22]	0.810	0.368
	C	0.158	0.182	1.18 [0.82 - 1.72]		
rs6330 *NGF*	GG	64 (34%)	65 (28.6%)	0.78 [0.51 - 1.18]	1.606	0.448
	AG	94 (50%)	119 (52.4%)	1.25 [0.8 - 1.93]		
	AA	30 (16%)	43 (18.9%)	1.41 [0.79 - 2.52]		
	G	0.590	0.548	0.84 [0.64 - 1.11]	1.476	0.224
	A	0.410	0.452	1.19 [0.9 - 1.56]		
rs3924999 *NRG1*	GG	75 (42.9%)	82 (35.8%)	0.74 [0.5 - 1.11]	3.587	0.166
	AG	88 (50.3%)	121 (52.8%)	1.26 [0.83 - 1.91]		
	AA	12 (6.9%)	26 (11.4%)	1.98 [0.93 - 4.2]		
	G	0.680	0.622	0.78 [0.58 - 1.04]	2.896	0.089
	A	0.320	0.378	1.29 [0.96 - 1.73]		

AD - patients with affective disorder; HC - healthy control; OR [95% CI] - Odds Ratios and 95% Confidence Intervals; SNP - Single Nucleotide Polymorphism; χ2 - chi-square criterion; * - statistical significance $p < 0.05$.

Table S2. Association of polymorphic variant rs7124442 of *BDNF* gene with clinical characteristics of the affective disorders.

Psychometric scales	CC	CT	TT	χ2	p
HARS upon admission	15.5 (11.25 : 26.5)	17 (11 : 26)	19 (14 : 26)	3.610	0.164
HARS on day 14 of therapy	14.5 (9.25 : 19.5)	7 (4.5 : 12.5)	10 (7 : 15)	9.710	**0.008***
HARS on day 28 of therapy	5.5 (2.75 : 6)	3 (2 : 7)	4 (2 : 7)	1.110	0.574
SIGH-SAD typical depressive symptoms upon admission	21 (13.25 : 25)	20 (16 : 25)	21 (17 : 27)	2.578	0.275
SIGH-SAD atypical depressive symptoms upon admission	9 (4.5 : 12.75)	6 (4 : 8)	6 (4 : 9)	0.847	0.655
SIGH-SAD total upon admission	29.5 (19.75 : 37.75)	28 (22 : 32)	29 (22.5 : 36)	1.928	0.381
SIGH-SAD typical depressive symptoms on day 14 of therapy	13 (10.25 : 15.75)	9 (5.25 : 12)	11 (8 : 14)	7.893	**0.019***
SIGH-SAD atypical depressive symptoms on day 14 of therapy	4.5 (1.75 : 7)	4 (2 : 6)	3 (2 : 5)	0.226	0.893
SIGH-SAD total on day 14 of therapy	17 (13 : 21.25)	14 (8.5 : 17)	14 (12 : 20)	4.147	0.126
SIGH-SAD typical depressive symptoms on day 28 of therapy	6 (4.25 : 7.75)	4 (2 : 7)	4 (2 : 7)	2.286	0.319

SIGH-SAD atypical depressive symptoms on day 28 of therapy	3.5 (1.5 : 5.5)	2 (0.75 : 3)	2 (0 : 3)	3.467	0.177
SIGH-SAD total on day 28 of therapy	11 (6.5 : 11)	6 (3 : 9)	6 (3 : 10)	2.508	0.285
CGI-S upon admission	4.5 (4 : 5.75)	4 (4 : 4)	4 (4 : 4.75)	3.923	0.141
CGI-S on day 14 of therapy	3 (3 : 5)	3 (3 : 3)	3 (3 : 4)	3.832	0.147
CGI-I on day 14 of therapy	3 (3 : 3)	3 (2 : 3)	3 (2 : 3)	3.736	0.154
CGI-S on day 28 of therapy	2.5 (2 : 3)	2 (2 : 2)	2 (2 : 3)	5.254	**0.072**
CGI-I on day 28 of therapy	2 (2 : 2)	2 (1 : 2)	2 (1 : 2)	1.591	0.451

$\chi 2$ - chi-square criterion; * - statistical significance $p < 0.05$.

Table S3. Association of polymorphic variant rs7103411 of *BDNF* with clinical characteristics of the affective disorders.

**Psychometric scales**	**CC**	**CT**	**TT**	**$\chi 2$**	**p**
HARS upon admission	33 (13 : 34.5)	17.5 (13 : 24)	18 (13 : 27)	1.200	0.549
HARS on day 14 of therapy	11 (6.25 : 20.25)	9 (5.75 : 13.25)	9 (6 : 13.5)	0.443	0.801
HARS on day 28 of therapy	7 (3 : 11)	4 (2 : 7)	4 (2 : 7)	1.740	0.419
SIGH-SAD typical depressive symptoms upon admission	20 (17 : 25.5)	20.5 (16 : 26)	21 (17 : 26)	0.200	0.905
SIGH-SAD atypical depressive symptoms upon admission	7 (5.5 : 9.5)	6.5 (4 : 8.25)	6 (4 : 10)	0.337	0.845
SIGH-SAD total upon admission	28.5 (22.75 : 38.75)	28 (21 : 35)	29 (23 : 34.5)	0.355	0.837
SIGH-SAD typical depressive symptoms on day 14 of therapy	9 (7.25 : 10.75)	11 (7.5 : 14)	10 (7 : 13)	0.853	0.653
SIGH-SAD atypical depressive symptoms on day 14 of therapy	5 (3.25 : 6)	4 (2.5 : 5)	3 (2 : 6)	0.734	0.693
SIGH-SAD total on day 14 of therapy	14 (12 : 14)	13 (10 : 18.25)	14 (10 : 18)	0.138	0.933
SIGH-SAD typical depressive symptoms on day 28 of therapy	5 (4 : 6.5)	5 (2 : 7.5)	4 (2 : 7)	2.212	0.331
SIGH-SAD atypical depressive symptoms on day 28 of therapy	2 (2 : 3)	2 (1 : 3)	2 (0 : 3)	1.944	0.378
SIGH-SAD total on day 28 of therapy	7 (6.5 : 9)	6.5 (3 : 11)	6 (3 : 10)	2.185	0.335
CGI-S upon admission	4 (4 : 4.5)	4 (4 : 5)	4 (4 : 4)	0.168	0.919
CGI-S on day 14 of therapy	3 (3 : 3)	3 (3 : 3)	3 (3 : 4)	0.369	0.832
CGI-I on day 14 of therapy	3 (3 : 3)	3 (2 : 3)	3 (2 : 3)	1.335	0.513
CGI-S on day 28 of therapy	2 (2 : 3.5)	2 (2 : 3)	2 (2 : 2)	5.723	**0.057**
CGI-I on day 28 of therapy	2 (1.5 : 2.5)	2 (1 : 2)	2 (1 : 2)	0.832	0.660

$\chi 2$ - chi-square criterion; * - statistical significance $p < 0.05$.

Table S4. Association of polymorphic variant rs11030104 of *BDNF* with clinical characteristics of the affective disorders.

**Psychometric scales**	**AA**	**AG**	**GG**	**$\chi 2$**	**p**
HARS upon admission	18 (12 : 27)	17 (13 : 24)	24 (12 : 35.25)	0.607	0.738
HARS on day 14 of therapy	9 (6 : 13)	9 (5 : 13)	7 (6 : 15)	0.025	0.988
HARS on day 28 of therapy	4 (2 : 7)	4 (2 : 7)	5 (3 : 10)	0.449	0.799
SIGH-SAD typical depressive symptoms upon admission	21 (17 : 26)	19.5 (16 : 25.75)	18.5 (17 : 20.75)	1.007	0.604
SIGH-SAD atypical depressive symptoms upon admission	6 (4 : 10)	6 (4 : 8)	6.5 (5.25 : 7.75)	0.093	0.954
SIGH-SAD total upon admission	29 (23 : 34.75)	28 (21 : 35.5)	25 (22 : 32)	1.000	0.606
SIGH-SAD typical depressive symptoms on day 14 of therapy	10 (7 : 13)	10 (7 : 14)	8 (7 : 10)	1.253	0.534
SIGH-SAD atypical depressive symptoms on day 14 of therapy	3 (2 : 6)	3 (2 : 5)	4 (3 : 6)	0.178	0.915
SIGH-SAD total on day 14 of therapy	14 (10.75 : 18)	13 (10 : 17.75)	13 (11.25 : 14)	1.301	0.522
SIGH-SAD typical depressive symptoms on day 28 of therapy	4 (2 : 7)	5 (2 : 8)	4.5 (4 : 5)	0.760	0.684
SIGH-SAD atypical depressive symptoms on day 28 of therapy	2 (0 : 3)	2 (1 : 3)	2 (2 : 2.75)	0.958	0.619
SIGH-SAD total on day 28 of therapy	6 (3 : 10)	6 (3 : 10.75)	7 (6.25 : 7.75)	0.731	0.694

CGI-S upon admission	4 (4 : 4.25)	4 (4 : 5)	4 (4 : 4)	0.095	0.953
CGI-S on day 14 of therapy	3 (3 : 4)	3 (3 : 3)	3 (3 : 3)	2.260	0.323
CGI-I on day 14 of therapy	3 (2 : 3)	3 (2 : 3)	3 (3 : 3)	1.361	0.506
CGI-S on day 28 of therapy	2 (2 : 2)	2 (2 : 3)	2 (2 : 2.75)	3.139	0.208
CGI-I on day 28 of therapy	2 (1 : 2)	2 (1 : 2)	2 (1.25 : 2)	0.126	0.939

χ2 - chi-square criterion; * - statistical significance $p < 0.05$.

Table S5. Association of polymorphic variant rs3924999 of *NRG1* with clinical characteristics of the affective disorders.

**Psychometric scales**	**AA**	**AG**	**GG**	**χ2**	**p**
HARS upon admission	14 (12 : 24)	18 (13 : 27)	19 (13.75 : 26.5)	2.057	0.358
HARS on day 14 of therapy	6 (4 : 13)	9 (6 : 14)	10 (6 : 13.5)	1.945	0.378
HARS on day 28 of therapy	4 (2.25 : 5.75)	4 (2 : 7)	4 (2 : 6.25)	0.638	0.727
SIGH-SAD typical depressive symptoms upon admission	16 (14 : 21.5)	21.5 (18 : 26)	21 (17 : 26)	7.969	**0.019***
SIGH-SAD atypical depressive symptoms upon admission	5.5 (4 : 8)	6 (4 : 8.75)	6.5 (4 : 10)	1.209	0.546
SIGH-SAD total upon admission	22 (20 : 32)	29 (23 : 35.75)	29.5 (23 : 34)	5.503	0.064
SIGH-SAD typical depressive symptoms on day 14 of therapy	8 (6 : 10)	11 (7 : 13.25)	11 (8 : 13)	4.537	0.103
SIGH-SAD atypical depressive symptoms on day 14 of therapy	4 (3 : 6)	3.5 (2 : 5)	3 (2 : 6)	2.321	0.313
SIGH-SAD total on day 14 of therapy	12.5 (10 : 16)	14 (10.5 : 18)	14 (10.25 : 18.75)	0.745	0.689
SIGH-SAD typical depressive symptoms on day 28 of therapy	4 (3 : 6)	4 (2 : 7)	4 (2 : 7)	0.010	0.995
SIGH-SAD atypical depressive symptoms on day 28 of therapy	2 (1 : 3)	1 (0 : 3)	2 (1 : 3)	1.568	0.457
SIGH-SAD total on day 28 of therapy	6 (4 : 8)	6 (3 : 10)	7 (3 : 11)	0.385	0.825
CGI-S upon admission	4 (4 : 5)	4 (4 : 4)	4 (4 : 5)	1.475	0.478
CGI-S on day 14 of therapy	3 (3 : 4)	3 (3 : 3)	3 (3 : 4)	2.347	0.309
CGI-I on day 14 of therapy	3 (2.25 : 3)	3 (2 : 3)	3 (2 : 3)	2.107	0.349
CGI-S on day 28 of therapy	2 (2 : 3)	2 (2 : 2)	2 (2 : 3)	3.380	0.184
CGI-I on day 28 of therapy	2 (2 : 2)	2 (1 : 2)	2 (2 : 2)	9.680	**0.008***

χ2 - chi-square criterion; * - statistical significance $p < 0.05$.

Table S6. Association of polymorphic variant rs6330 of *NGF* with clinical characteristics of the affective disorders.

**Psychometric scales**	**AA**	**AG**	**GG**	**χ2**	**p**
HARS upon admission	19 (12 : 25)	19 (13 : 26)	17 (12.75 : 27.25)	0.054	0.973
HARS on day 14 of therapy	10 (6 : 15)	9 (5 : 14)	8.5 (6 : 13.25)	0.294	0.863
HARS on day 28 of therapy	5 (2 : 6)	3 (2 : 7)	4.5 (2.75 : 7)	1.568	0.456
SIGH-SAD typical depressive symptoms upon admission	20 (16 : 25)	21 (17 : 26)	21 (16 : 25)	0.991	0.609
SIGH-SAD atypical depressive symptoms upon admission	6 (4 : 11)	6 (4 : 9)	6 (4 : 8)	1.051	0.591
SIGH-SAD total upon admission	29 (24.5 : 33.5)	30 (22 : 36)	28 (22 : 33.5)	2.123	0.346
SIGH-SAD typical depressive symptoms on day 14 of therapy	12 (8 : 14)	11 (8 : 14)	10 (6.75 : 12)	4.048	0.132
SIGH-SAD atypical depressive symptoms on day 14 of therapy	4 (3 : 5)	4 (2 : 6)	3 (2 : 4.25)	3.337	0.188
SIGH-SAD total on day 14 of therapy	15 (12.25 : 18)	14 (10.5 : 18.75)	13 (9 : 15)	5.167	**0.076**
SIGH-SAD typical depressive symptoms on day 28 of therapy	4 (2 : 7.25)	4.5 (2 : 7)	4 (2 : 6.5)	0.808	0.668
SIGH-SAD atypical depressive symptoms on day 28 of therapy	2 (1 : 3)	2 (0 : 3)	2 (1 : 2.5)	0.789	0.674
SIGH-SAD total on day 28 of therapy	7 (3 : 11)	6 (3 : 11)	6 (3 : 9)	1.391	0.499
CGI-S upon admission	4 (4 : 4)	4 (4 : 5)	4 (4 : 5)	0.806	0.668
CGI-S on day 14 of therapy	3 (3 : 3)	3 (3 : 4)	3 (3 : 4)	0.262	0.877
CGI-I on day 14 of therapy	3 (2.25 : 3)	3 (2 : 3)	3 (2 : 3)	1.795	0.408

CGI-S on day 28 of therapy	2 (2 : 2)	2 (2 : 3)	2 (2 : 3)	2.260	0.323
CGI-I on day 28 of therapy	2 (1 : 2)	2 (1 : 2)	2 (1 : 2)	0.003	0.999

$\chi 2$ - chi-square criterion; * - statistical significance $p < 0.05$.
